# Diagnosis of ventilator-associated pneumonia using electronic nose sensor array signals: solutions to improve the application of machine learning in respiratory research

**DOI:** 10.1186/s12931-020-1285-6

**Published:** 2020-02-07

**Authors:** Chung-Yu Chen, Wei-Chi Lin, Hsiao-Yu Yang

**Affiliations:** 1 Division of Pulmonary and Critical Care Medicine, Department of Internal Medicine, National Taiwan University Hospital Yunlin Branch, Douliu, Taiwan; 2 Institute of Occupational Medicine and Industrial Hygiene, National Taiwan University College of Public Health, Taipei, Taiwan; 3 Institute of Environmental and Occupational Health Sciences, National Taiwan University College of Public Health, Taipei, Taiwan; 4 Department of Public Health, National Taiwan University College of Public Health, Taipei, Taiwan; 5 Department of Environmental and Occupational Medicine, National Taiwan University Hospital, Taipei, Taiwan; 6 Innovation and Policy Center for Population Health and Sustainable Environment, College of Public Health, National Taiwan University, Taipei, Taiwan

**Keywords:** Electronic nose, Breath test, Machine learning, Ventilator-associated pneumonia, Volatile organic compounds

## Abstract

**Background:**

Ventilator-associated pneumonia (VAP) is a significant cause of mortality in the intensive care unit. Early diagnosis of VAP is important to provide appropriate treatment and reduce mortality. Developing a noninvasive and highly accurate diagnostic method is important. The invention of electronic sensors has been applied to analyze the volatile organic compounds in breath to detect VAP using a machine learning technique. However, the process of building an algorithm is usually unclear and prevents physicians from applying the artificial intelligence technique in clinical practice. Clear processes of model building and assessing accuracy are warranted. The objective of this study was to develop a breath test for VAP with a standardized protocol for a machine learning technique.

**Methods:**

We conducted a case-control study. This study enrolled subjects in an intensive care unit of a hospital in southern Taiwan from February 2017 to June 2019. We recruited patients with VAP as the case group and ventilated patients without pneumonia as the control group. We collected exhaled breath and analyzed the electric resistance changes of 32 sensor arrays of an electronic nose. We split the data into a set for training algorithms and a set for testing. We applied eight machine learning algorithms to build prediction models, improving model performance and providing an estimated diagnostic accuracy.

**Results:**

A total of 33 cases and 26 controls were used in the final analysis. Using eight machine learning algorithms, the mean accuracy in the testing set was 0.81 ± 0.04, the sensitivity was 0.79 ± 0.08, the specificity was 0.83 ± 0.00, the positive predictive value was 0.85 ± 0.02, the negative predictive value was 0.77 ± 0.06, and the area under the receiver operator characteristic curves was 0.85 ± 0.04. The mean kappa value in the testing set was 0.62 ± 0.08, which suggested good agreement.

**Conclusions:**

There was good accuracy in detecting VAP by sensor array and machine learning techniques. Artificial intelligence has the potential to assist the physician in making a clinical diagnosis. Clear protocols for data processing and the modeling procedure needed to increase generalizability.

## Background

Ventilator-associated pneumonia (VAP) is a significant cause of mortality, and the most common nosocomial infection in the intensive care unit (ICU) [1]. According to the US National Nosocomial Infection Surveillance system, one-third of all nosocomial infections in ICUs are pneumonia; of these, 83% are associated with mechanical ventilation [2]. Common pathogens of VAP include *Pseudomonas aeruginosa*, *Staphylococcus aureus*, methicillin-resistant *Staphylococcus aureus*, *Klebsiella pneumoniae*, and *Acinetobacter baumannii* [3]. Patients who acquire VAP have poorer outcomes, higher mortality rates, and longer lengths of hospital stay than uninfected patients [4]. Delays in the initiation of appropriate antibiotic treatment for VAP significantly increase mortality [5]. Patients with suspected VAP should undergo a serial evaluation that includes chest X-ray, sputum Gram stain, sputum cultures, and blood cultures. The sputum Gram stain is not reliable for the early application of antibiotic therapy. Sputum culture requires a long culture duration, and the results might not correlate well with the real causative pathogen [6]. Mechanically ventilated (MV) patients usually empirically receive broad-spectrum antibiotics to control a suspected infection at the earliest time, which may result in the development of resistance [7]. Early diagnosis for VAP is important to provide appropriate treatment and reduce mortality. However, current culture-based microbiological diagnosis is inadequate for the timely prescription of targeted antibiotics. The development of a rapid test to diagnose VAP is therefore important to solve these problems [8].

Many bacterial species can produce volatile metabolites via catabolic pathways, including glycolysis, proteolysis, and lipolysis [9]. According to gas chromatography/mass spectrometry (GC-MS) analysis, as many as 34 volatile metabolites were released from *Streptococcus pneumoniae* and 28 from *Haemophilus influenzae* in vitro, comprising alcohols, aldehydes, esters, hydrocarbons, ketones, and sulfur-containing compounds [10]. In an animal study, the volatile organic compounds (VOCs) released from the breath of mice with lung infections of *Pseudomonas aeruginosa* and *Staphylococcus aureus* were also detectable in cultures in vitro [11]. These findings were also noted in some human studies conducted in VAP with *Staphylococcus aureus, Escherichia coli, Candida albicans,* and *Acinetobacter baumannii* infection [12, 13]. The findings suggested that discrimination of the VOCs derived from pathogens might provide a noninvasive breath test for the diagnosis of VAP.

A novel sensor array technique has been developed to discriminate the VOCs in breath [14]. The estimated number of metabolites in humans ranges from a low of 2000–3000 to a high of approximately 20,000 [15]. It is difficult to qualitatively and quantitatively measure all the VOCs by GC-MS because most of the compounds are still unknown. To discriminate the VOCs associated with diseases, there is increasing interest in using an electronic nose to address the problem [14]. An electronic nose uses sensor responses to measure VOCs. During the measurements, the VOCs attach to the sensor polymer surface to induce swelling of the polymer film. The swelling increases the electrical resistance of the composite, which generates an electrical signal. The sensor array response data are subsequently used as predictors to create a diagnostic classification algorithm [16]. Artificial intelligence (AI) has been gradually used in medicine to assist physicians in making clinical diagnoses [17]. Machine learning technology is commonly used in the analysis of sensor response data because an electronic nose is a composite of a sensor array and functionally resembles biological olfactory receptors by pattern recognition. However, the process of building algorithms is usually a “black box”, and the results are over optimized in many studies, preventing physicians from applying them in clinical practice. Clear processes of model building and assessing accuracy are therefore warranted. The objective of this study was to use sensor array signals to diagnose VAP using the machine learning technique. Using this study as an example, we demonstrated our procedures to build machine learning algorithms and assess their accuracy for facilitating the application of AI in medicine.

## Methods

### Study subjects

We recruited cases of VAP and ventilated controls without VAP in the ICU of National Taiwan University Hospital Yunlin Branch. The diagnosis of VAP was based on three components: clinical signs of infection (fever, increased white blood cell counts, or purulent tracheobronchial secretions); new or worsening infiltrates on a chest X-ray; and bacteriologic evidence of pulmonary parenchymal infection [18].

### Microbiological report

The microbiological report of VAP was based on the culture of lower respiratory tract secretions obtained from the endotracheal aspirate, tracheostomy tube suction, or bronchoscopy. Lower respiratory tract secretions were obtained before antibiotics were started or changed.

### Medical history and examination

Physicians obtained a medical history from the medical records. All subjects received a chest X-ray, a complete blood count (CBC), a blood urine nitrogen (BUN), a creatinine, a fasting sugar, an aspartate aminotransferase (AST), an alanine aminotransferase (ALT), and a urinary analysis. We obtained a cigarette smoking history from the medical records or his/her family.

### Diagnosis of VAP

The diagnosis of VAP was ascertained by a pulmonologist and an infectious disease physician using clinical signs/symptoms, laboratory reports, and chest X-rays. The pathogens of pneumonia were confirmed by culture of lower respiratory tract secretions. The study subjects and physicians were blinded to the results of an electronic nose analysis.

### Breath air sampling

VOCs generated by causative pathogens of VAP are best collected in the lower respiratory tract. We collected alveolar air from an endotracheal tube to prevent contamination from environmental air, the oral cavity, and dead space air and to increase the concentration of VOCs derived from pathogens [19]. To prevent contamination from food, the sampling was performed before feeding. We collected a 1-l volume of alveolar air in a Tedlar Bag (SKC, Inc., USA). A new bag was used to prevent potential contamination from incomplete cleaning of the reused bags.

### Electronic nose analysis

The electronic nose analysis followed our standardized procedure [20]. In brief, the collected air was sent back to the laboratory for analysis within 48 h. The air was analyzed using the Cyranose 320 electronic nose (Sensigent, CA, USA), which has 32 thin-film nanocomposite sensors. For each of the 32 sensors, ten consecutive measurements from the same breath were collected to obtain a mean value for analysis after the deletion of the first measurements according to the manufacturer’s suggestion [21]. Because the expiratory flow rate would significantly affect the measurement [22], a constant flow rate of 120 cc/min was standardized for all measurements. The Cyranose 320 uses conducting polymer arrays, which might be influenced by the temperature of the sample gas. Therefore, we maintained a constant temperature of 20–22 degrees Celsius during all analyses. We analyzed all samples in the same room with a fixed temperature and humidity. The room air pumped into the electronic nose was analyzed to provide the baseline sensor response (R_0_). The raw data were normalized and autoscaled to eliminate background noise and exclude outliers [22, 23] and then used to derive the prediction model.

1 $$ \mathrm{Sensor}\ \mathrm{response}:\frac{\Delta \mathrm{R}}{R_o}=\frac{\left({R}_{max}-{R}_0\right)}{R_0}. $$

The raw data were normalized using the equation

2 $$ \sum \limits_{k=1}^{\mathrm{NV}}{x}_{ik}^2={c}_i $$

where *k* designates the sensor, *i* designates the gas, and NV is the total number of sensors. Then, the data were autoscaled to the unit variance that refers to mean centering and then divided by the standard deviation:

3 $$ {x}_{ik}^{\hbox{'}}=\frac{x_{ik}-{\overline{x}}_k}{s_k} $$

where ${x}_{ik}^{\hbox{'}}$ is the autoscaled response, *x*_*ik*_ is the relative sensor response, ${\overline{x}}_k$ is the mean value of the normalized response for the specific sensor and *s*_*k*_ is the standard deviation.

4 $$ {s}_k={\left[\frac{1}{\mathrm{NP}-1}\sum \limits_{i=1}^{\mathrm{NP}}{\left({x}_{ik}-{\overline{x}}_k\right)}^2\right]}^{1/2} $$

Autoscaling removes any inadvertent weighting that arises due to arbitrary units. After autoscaling, the value distribution of each sensor across the entire database was set to a mean value of zero and unit standard deviation [23].

### Statistical analysis

**Fig. 1:**
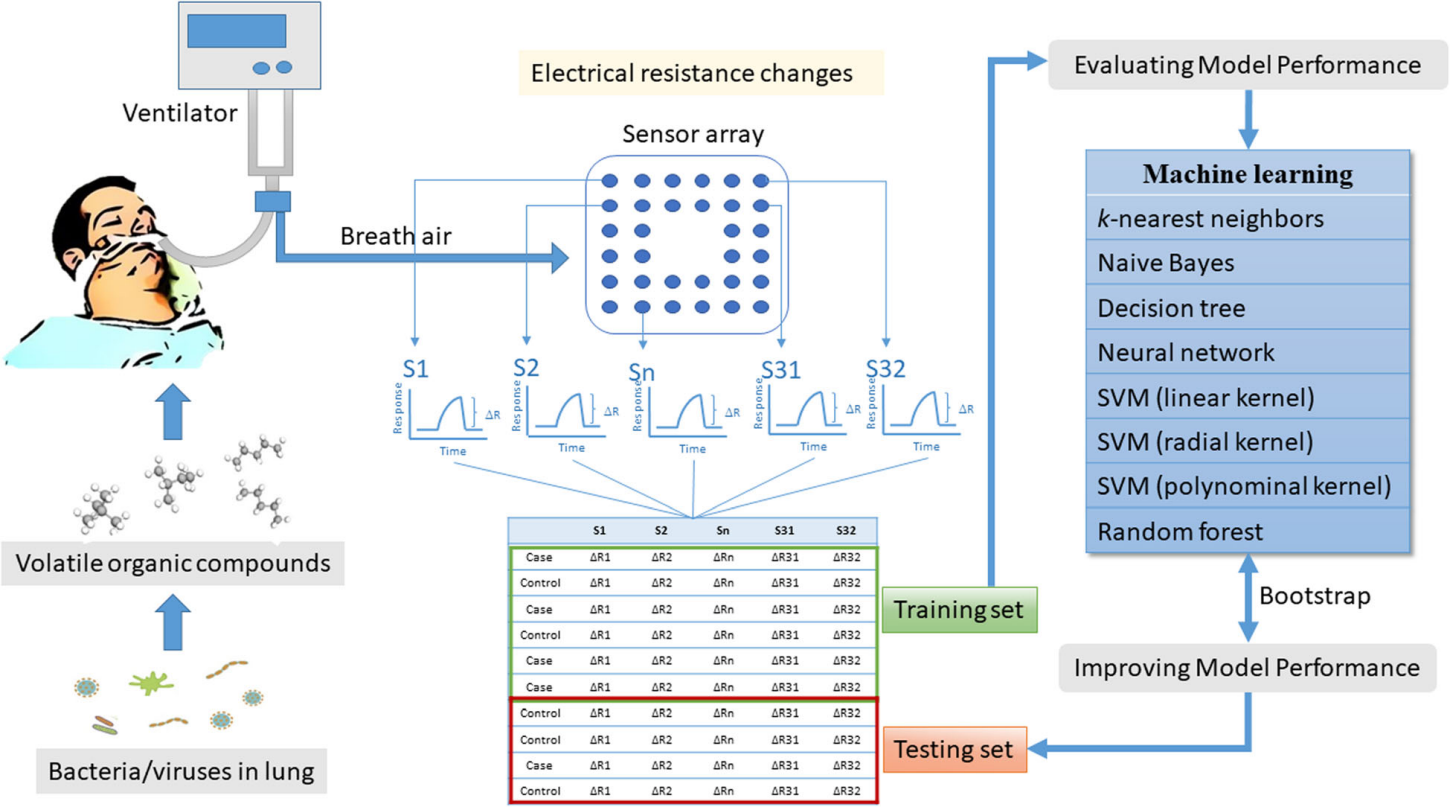
Flow diagram of this study. The diagram shows our standardized procedures of data collection, data preparation, model building, model evaluation, and model improvement. When the pathogens are colonized in the lung, they will release volatile organic compounds in the breath. We collected the breath from the endotracheal tube and then analyzed the sensor arrays of an electronic nose. The electric resistance changes of sensors were first normalized and autoscaled. Then, we randomly split subjects into a training set and a testing set. We used eight machine learning algorithms to estimate diagnostic accuracy. The parameters of the algorithms are selected with bootstrapping methods. The optimized models were then applied to the testing set to assess the accuracy of the breath test

We followed a standardized protocol of establishing machine learning algorithms with a five-step process, namely, data collection, data preparation, model building, model evaluation, and model improvement (Fig. 1). We planned the analytical protocols before the study was performed. We randomly split data into a training set (80%) for model derivation and a testing set (20%) for validation. The training set was used to generate the model. We used the modelLookup function of the caret package for automated parameter tuning to improve model performance [24]. Then, the optimized models were further tested in an independent testing set to evaluate the accuracy. To prevent unequal distribution in the proportion of cases in each group, we applied the oversampling method that replicates the observations a from minority class to balance the data [25]. Using the confusion matrix, we determined the accuracy, sensitivity, specificity, positive prediction rate, and negative prediction value [26]. In this study, we used eight machine learning algorithms to establish the prediction models, including *k*-nearest neighbors, Naive Bayes, decision tree, neural network, support vector machines (SVMs) (including linear kernel, polynomial kernel, and radial basis kernel), and random forest.

### K-nearest neighbors

The *k*-nearest neighbors algorithm uses information about an example’s *k*-nearest neighbors to classify unlabeled examples by calculating the distance between two points. We used the Euclidean distance by the following formula:

5 $$ \mathrm{dist}\left(\mathrm{p},\mathrm{q}\right)=\sqrt{{\left({\mathrm{p}}_1-{\mathrm{q}}_1\right)}^2+{\left({\mathrm{p}}_2-{\mathrm{q}}_2\right)}^2+...+{\left({\mathrm{p}}_{\mathrm{n}}-{\mathrm{q}}_{\mathrm{n}}\right)}^2} $$

where p and q are the examples to be compared, each having n features. The strength of *k*-nearest neighbors is that it makes no assumptions about the underlying data distribution [27]. We used the R package “class” to build the *k*-nearest neighbors model [28].

### Naive Bayes

The Naive Bayes algorithm applied the Bayes’ theorem to classification, in which we can compute a posterior probability of an outcome event based on a prior probability:

6 $$ \mathrm{P}\left(\mathrm{A}|\mathrm{B}\right)=\frac{\mathrm{P}\left(\mathrm{A}\cap \mathrm{B}\right)}{\mathrm{P}\left(\mathrm{B}\right)}=\frac{\mathrm{P}\left(\mathrm{B}|\mathrm{A}\right)\mathrm{P}\left(\mathrm{A}\right)}{\mathrm{P}\left(\mathrm{B}\right)} $$

The Naive Bayes classification algorithm can be summarized by the following algorithm:

7 $$ \mathrm{P}\left({\mathrm{C}}_{\mathrm{L}}|{\mathrm{F}}_1,....,{\mathrm{F}}_{\mathrm{n}}\right)=\frac{1}{Z}p\left({C}_L\right)\prod \limits_{i=1}^np\left({F}_i|{C}_L\right) $$

where the probability of level L for class C, given the evidence provided by features F_1_ through F_n_, is equal to the product of the probabilities of each piece of evidence conditioned on the class level, the prior probability of the class level, and a scaling factor, 1/Z, which converts the likelihood values into probabilities. The strength of Naive Bayes is that it requires relatively few examples for training and does well with noisy data. The weakness is that it assumes that all of the features in the database are equally important and independent, but the assumption is rarely true [27]. We used the R package “klaR” to build the Naive Bayes model [29].

### Decision tree

Decision tree utilizes a tree structure to model the relationships among the features and the potential outcomes. It uses entropy to quantify the randomness with a set of class values and find splits that reduce entropy. Entropy is specified as follows:

8 $$ \mathrm{Entropy}\left(\mathrm{S}\right)=\sum \limits_{\mathrm{i}=1}^c-{p}_i{\log}_2\left({p}_i\right) $$

for a given segment of data (S), term *c* refers to the number of class levels, and *p*_*i*_ refers to the proportion of values falling into class level *i*. Decision tree uses entropy to determine the optimal feature to split upon, and the algorithm calculates the change in homogeneity that would result from a split on each possible feature, which is a measure known as information gain. The information gain for a feature F is calculated as the difference between the entropy in the segment before the split (S_1_) and the partitions resulting from the split (S_2_):

9 $$ \mathrm{InfoGain}\left(\mathrm{F}\right)=\mathrm{Entropy}\left({\mathrm{S}}_1\right)-\mathrm{Entropy}\left({\mathrm{S}}_2\right) $$

Decision tree is best suited for tasks with many features or complexes and nonlinear relationships among features and outcomes [27]. We used the R package “C50” to build the decision tree model [30].

### Neural network

Artificial neural networks mimic the structure of animal brains to model arbitrary functions. The biological neuron that uses dendrites to receive a signal, and a neuron determines the importance of the signal and then decides whether to transmit the signal to the next neuron by the axon. The input signals are received by the dendrites (*x* variables), each dendrite’s signal is weighted (*w* values) according to its importance, and the output is the signal (*y* variable). The input signals are summed by the cell body, and the signal is passed on according to an activation function. With *n* input dendrites, the activation function can be represented by the following formula:

10 $$ {\displaystyle \begin{array}{l}y(x)=f\left(\sum \limits_{i=1}^n{w}_i{x}_i\right)\\ {}\end{array}} $$

where *n* refers to the number of input dendrites, and *w* weights allow each of the *n* inputs (denoted by *x*_*i*_) to contribute a greater or lesser amount to the sum of input signals. The net total is used by the activation function f(*x*), and the resulting signal, *y*(*x*), is the output axon. The strength of neural networks is the capability to model complex patterns without making an assumption regarding the data’s underlying relationships. However, their weakness is that they are very prone to overfitting [27]. We used the R package “neuralnet” to build the neural network model [31].

### Support vector machines

SVMs creates a flat boundary called a hyperplane, which divides the space to create fairly homogeneous partitions on either side that allow SVMs to model highly complex relationships. SVMs uses a kernel trick to separate data into a higher dimension space. The kernel function is expressed as:

11 $$ \mathrm{K}\left(\overrightarrow{x_i},\overrightarrow{x_j}\right)=\varphi \left(\overrightarrow{x_i}\right)\times \varphi \left(\overrightarrow{x_j}\right) $$

where ϕ(x) is a function to transfer the feature vectors *x*_*i*_ and *x*_*j*_ and combine them into a single number. Using this form, kernel functions have been developed for many different domains of data. The linear kernel does not transform the data at all. The polynomial kernel of degree *d* adds a simple nonlinear transformation of the data. The radial basis kernel is similar to a neural network and can perform well on many types of data [27].

12 $$ {\displaystyle \begin{array}{l}\mathrm{Linear}\ \mathrm{kernel}:\kern0.6em \mathrm{K}\left(\overrightarrow{x_i},\overrightarrow{x_j}\right)=\overrightarrow{x_i}\times \overrightarrow{x_j}\\ {}\mathrm{Polynomial}\ \mathrm{kernel}:\kern0.6em \mathrm{K}\left(\overrightarrow{x_i},\overrightarrow{x_j}\right)={\left(\overrightarrow{x_i}\times \overrightarrow{x_j}+1\right)}^d\\ {}\mathrm{Radial}\ \mathrm{basis}\ \mathrm{kernel}:\kern0.6em \mathrm{K}\left(\overrightarrow{xi},\overrightarrow{xj}\right)={e}^{\frac{-{\left\Vert \overrightarrow{x_i}-\overrightarrow{x_j}\right\Vert}^2}{2{o}^2}}\end{array}} $$

We used the R package “kernlab” to build the SVMs model [32].

### Random forest

A random forest is an ensemble consisting of random trees, which are decision trees generated in a specific way to obtain diversity among the trees [33]. The strength of a random forest is the ability to handle an extremely large number of features or examples that are easy to use [27]. We created an ensemble of 500 trees and used the out-of-bag error rate to estimate the test set error [34]. We used the R package “randomForest” to build the random forest model [35].

### Accuracy

Using the physicians’ clinical diagnosis as the golden standard for VAP, we assessed the performance of all these methods based on accuracy, the area under the receiver operator characteristic (ROC) curves (AUC), sensitivity, specificity, accuracy and kappa value. AUC values of 0.7–0.8, 0.8–0.9, and 0.9–1 are regarded as good, very good, and excellent diagnostic accuracy, respectively [36]. To adjust accuracy by accounting for the possibility of a correct prediction by chance only, which is especially important for datasets with class imbalance, we also calculated the kappa statistics [27] Kappa expresses the extent to which the observed agreement exceeds that which would be expected by chance alone. A kappa greater than 0.75 represents excellent agreement beyond chance, a kappa below 0.40 represents poor agreement, and a kappa of 0.40 to 0.75 represents intermediate to good agreement. We used a bootstrap method and calculated the accuracy of 100 iterations to decide the parameters of machine learning methods that had the highest prediction accuracy. The statistics can be defined by the following equations:

13 $$ {\displaystyle \begin{array}{l}\mathrm{Accuracy}=\frac{\mathrm{Number}\ \mathrm{of}\ \mathrm{true}\ \mathrm{positives}+\mathrm{number}\ \mathrm{of}\ \mathrm{true}\ \mathrm{negatives}}{\mathrm{Number}\ \mathrm{of}\ \mathrm{true}\ \mathrm{positives}+\mathrm{true}\ \mathrm{negatives}+\mathrm{false}\ \mathrm{positives}+\mathrm{false}\ \mathrm{negatives}}\\ {}\mathrm{Sensitivity}=\frac{\mathrm{Number}\ \mathrm{of}\ \mathrm{true}\ \mathrm{positives}\ }{\mathrm{Number}\ \mathrm{of}\ \mathrm{true}\ \mathrm{positives}+\mathrm{Number}\ \mathrm{of}\ \mathrm{false}\ \mathrm{negatives}}\\ {}\mathrm{Specificity}=\frac{\mathrm{Number}\ \mathrm{of}\ \mathrm{true}\ \mathrm{negatives}}{\mathrm{Number}\ \mathrm{of}\ \mathrm{true}\ \mathrm{negatives}+\mathrm{Number}\ \mathrm{of}\ \mathrm{false}\ \mathrm{positives}\ }\\ {}\mathrm{Positive}\ \mathrm{predictive}\ \mathrm{value}=\frac{\mathrm{Number}\ \mathrm{of}\ \mathrm{true}\ \mathrm{positives}}{\mathrm{Number}\ \mathrm{of}\ \mathrm{true}\ \mathrm{positives}+\mathrm{Number}\ \mathrm{of}\ \mathrm{false}\ \mathrm{positives}}\\ {}\mathrm{Negative}\ \mathrm{predictive}\ \mathrm{value}=\frac{\mathrm{Number}\ \mathrm{of}\ \mathrm{true}\ \mathrm{negatives}}{\mathrm{Number}\ \mathrm{of}\ \mathrm{true}\ \mathrm{negatives}+\mathrm{Number}\ \mathrm{of}\ \mathrm{false}\ \mathrm{negatives}}\\ {}\mathrm{Kappa}=\frac{\left(\mathrm{Percent}\ \mathrm{agreement}\ \mathrm{observed}\right)-\left(\mathrm{Percent}\ \mathrm{agreement}\ \mathrm{expected}\ \mathrm{by}\ \mathrm{chance}\ \mathrm{alone}\right)}{100\%-\left(\mathrm{Percent}\ \mathrm{agreement}\ \mathrm{expected}\ \mathrm{by}\ \mathrm{chance}\ \mathrm{alone}\right)}\end{array}} $$

We also constructed ROC curves and calculated an AUC with 2000 bootstrap replicates and the partial AUC (pAUC) to assess the variability of the measure. The formula of pAUC was:

14 $$ pROC=\frac{1}{2}\left(1+\frac{pAUC-\min }{\max -\min}\right), $$

where *min* is the pAUC over the same region of the diagonal ROC curve, and *max* is the pAUC over the same region of the perfect ROC curve [37]. Because we were interested in a diagnostic test with a high specificity and sensitivity, we also examined the partial AUC between 80 and 100% for specificity and sensitivity.

## Results

A total of 61 subjects were enrolled. After excluding two subjects without a questionnaire, a total of 33 cases and 26 controls were used in the final analysis. In the case group, the primary pathogen in sputum culture was most commonly *Klebsiella pneumoniae* (42.42%), followed by *Stenotrophomonas maltophilia* (15.15%), *Staphylococcus aureus* (15.15%), *Acinetobacter baumannii* (12.12%), *Pseudomonas aeruginosa* (12.12%), *Escherichia coli* (6.06%), *Candida albicans* (6.06%), *Haemophilus influenzae* (3.03%), and *Enterobacter cloacae* complex (3.03%). The mean number of the pathogens was 1.52. In addition to pneumonia, the comorbidities in the case group included myocardial infarction, diabetes, aspiration pneumonia, hepatitis, endocarditis, heart failure, lung cancer, chronic obstructive pulmonary disease, hepatocellular carcinoma, idiopathic pulmonary fibrosis, colon cancer, necrotizing fasciitis, kidney injuries, hyponatremia, and cardiac arrest. In the control group, the comorbidities included intracranial hemorrhage, gastric cancer, traffic accident, fracture, gastric ulcer, coronary artery disease, acute kidney injury, traumatic brain injury, aortic dissection, lung cancer, Fournier’s gangrene, and liver abscess. There was no statistically significant difference in age, gender, smoking status, liver and renal function tests, or the number of comorbidities in both groups (Table 1).

**Table 1:** Demographic characteristics of the study subjects

Characteristics	Case group (*n* = 33)	Control group (*n* = 26)	*p* value
Age (year), mean (SD)	71.44 (13.43)	68.90 (15.55)	0.53
Male, No. (%)	21 (63.64)	13 (50.00)	0.12
Smoking status			0.18
Current smoker, No. (%)	4 (12.12)	4 (15.38)	
Former smoker, No. (%)	12 (36.36)	4 (15.38)	
Nonsmoker, No. (%)	15 (45.45)	12 (46.15)	
White blood cell (10^3^/μL), mean (SD)	14.63 (7.87)	15.03 (8.77)	0.86
Blood urea nitrogen (mg/dL), mean (SD)	30.65 (18.74)	32.54 (18.43)	0.72
Creatinine (mg/dL), mean (SD)	1.24 (0.71)	1.58 (1.01)	0.15
Aspartate aminotransferase (U/L), mean (SD)	88.48 (139.48)	53.71 (139.48)	0.25
Alanine aminotransferase (U/L), mean (SD)	55.80 (82.11)	35.62 (25.82)	0.22
Number of comorbidities	3.16 (1.10)	2.90 (1.18)	0.43

**Fig. 2:**
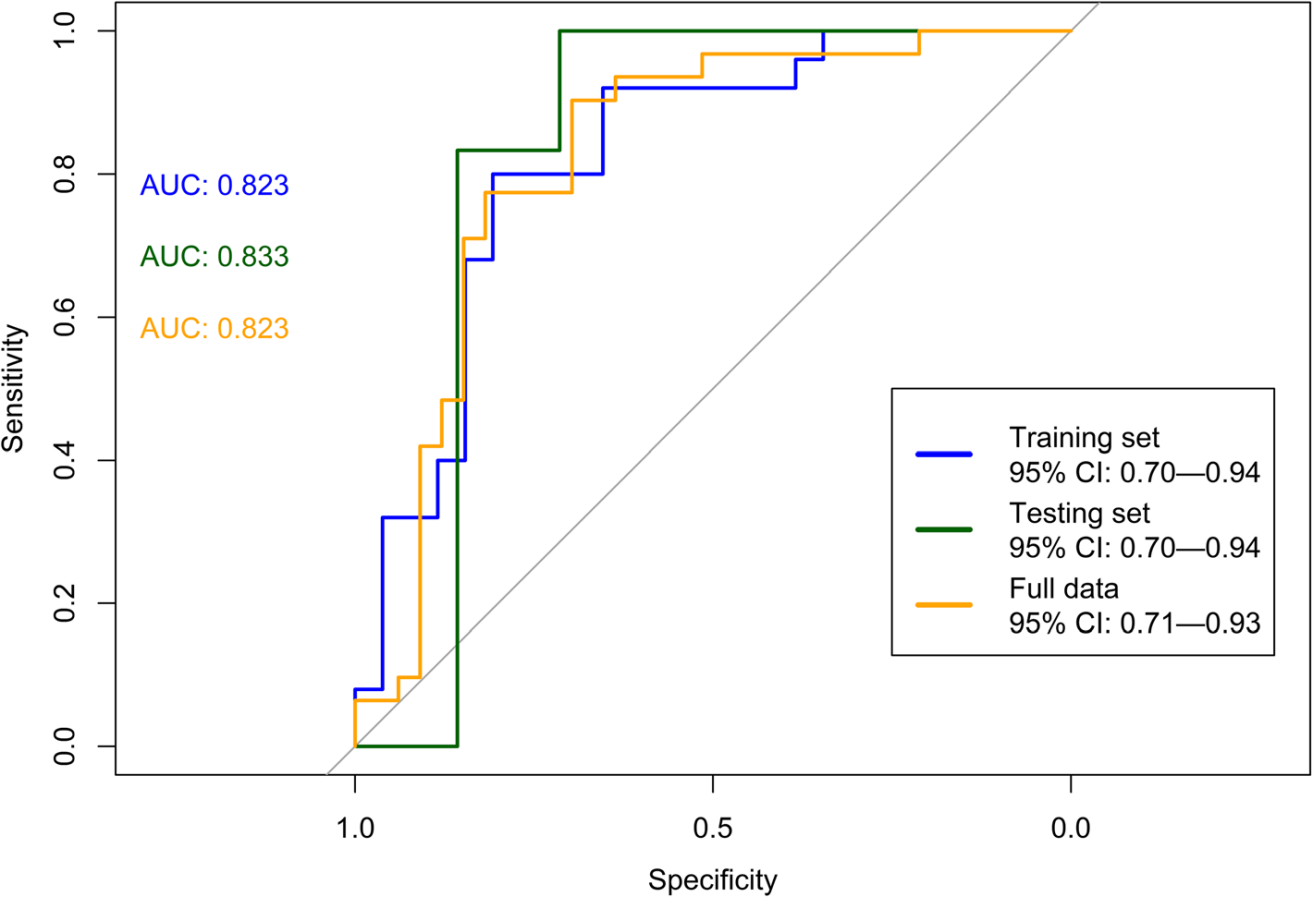
The area under the receiver operating curve (AUC) for ventilator-associated pneumonia in the training set, testing set, and full data set. High AUCs in the three sets show high diagnostic accuracy

**Fig. 3:**
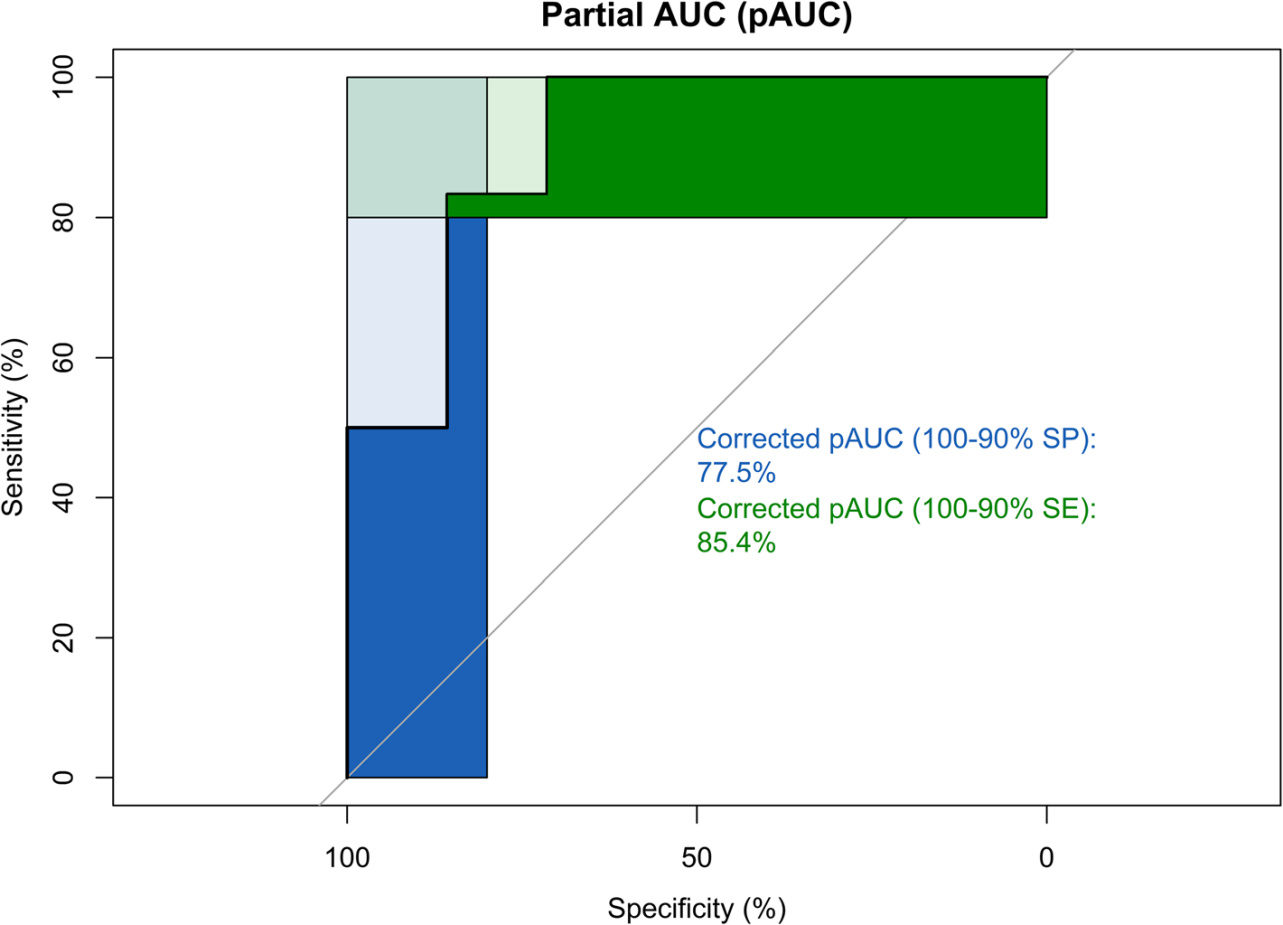
The partial area under the receiver operating curve (pAUC). The blue area corresponds to the pAUC region between 80 and 100% for specificity (SP), and the green area corresponds to the pAUC region between 80 and 100% for sensitivity (SE). The corrected pAUCs are printed in the middle of the plot

**Fig. 4:**
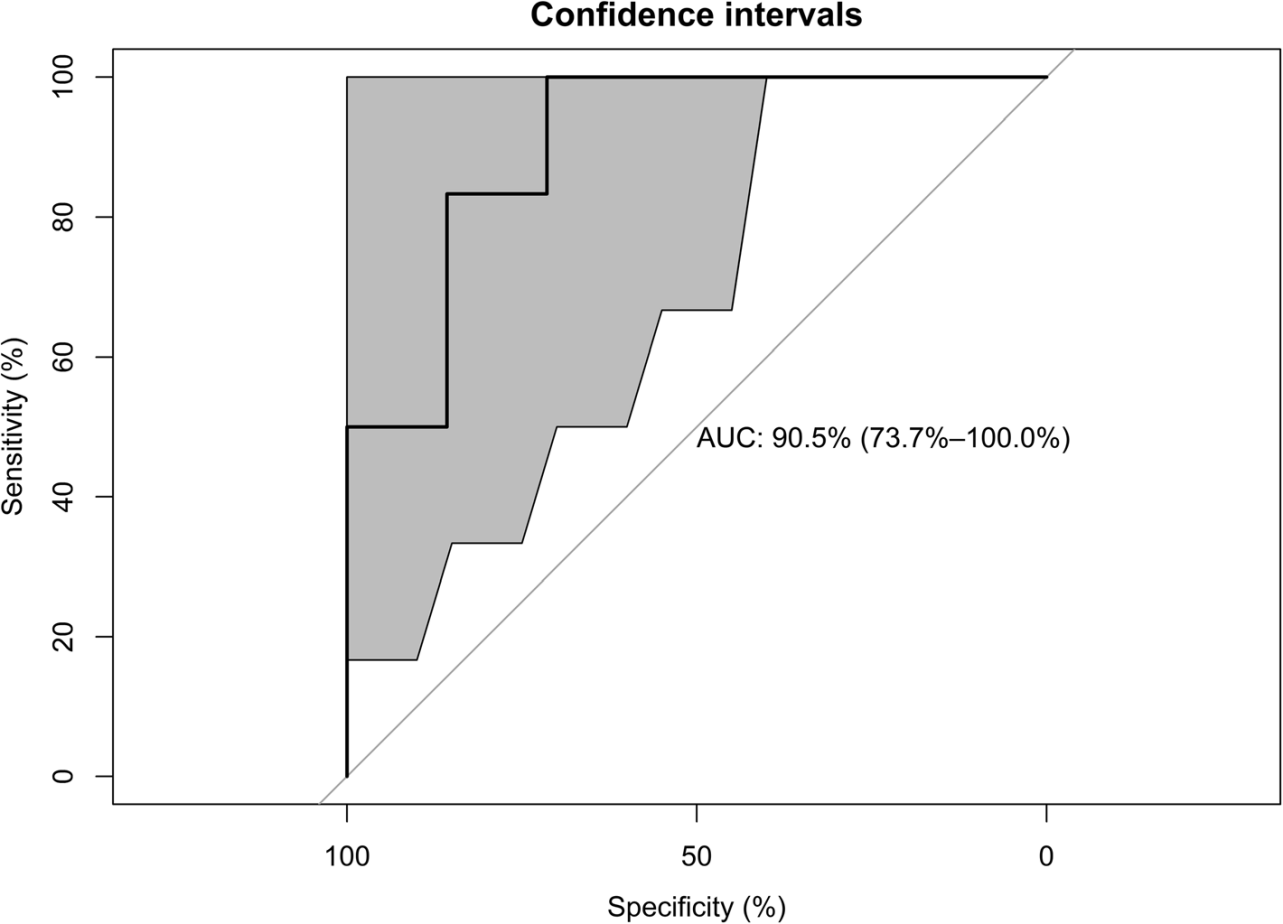
The area under the receiver operating curve (AUC) for ventilator-associated pneumonia in the testing set, with the 95% confidence interval. We used the random forest algorithm to establish the prediction model and then tested the prediction accuracy on the training set. Using bootstrap resampling for 2000 replicates, the 95% confidence intervals are shown as gray areas around the mean bootstrapped curve

Using eight machine learning algorithms, the mean accuracy in the testing set was 0.81 ± 0.04, the sensitivity was 0.79 ± 0.08, the specificity was 0.83 ± 0.00, the positive predictive value (PPV) was 0.85 ± 0.02, the negative predictive value (NPV) was 0.77 ± 0.06, and the AUC was 0.85 ± 0.04. The mean kappa value in the testing set was 0.62 ± 0.08, which suggested good agreement (Table 2). The AUCs were 0.82 (95% CI 0.70—0.94), 0.83 (0.70—0.94), and 0.82 (95% CI 0.71—0.93) in the training set, testing set, and the full data set, respectively (Fig. 2). In the testing set, the corrected pAUC between 80 and 100% for sensitivity was 85.4%. The corrected pAUC between 80 and 100% for specificity was 75.5% (Fig. 3). Using bootstrap resampling for 2000 replicates, the model established by the random forest algorithm had the highest AUC (Fig. 4).

**Table 2:** Prediction accuracy of the electronic nose in the test set of machine learning algorithms

Model and parameters	Accuracy (95% CI)	Sensitivity	Specificity	PPV	NPV	Kappa	AUC (95% CI)
*k*-nearest neighbors (*k* = 5)	0.77 (0.46–0.95)	0.71	0.83	0.83	0.71	0.54	0.80 (0.54–1.00)
Naive Bayes (fL = 0, usekernel = TRUE, adjust = 1)	0.77 (0.46–0.95)	0.71	0.83	0.83	0.71	0.54	0.80 (0.54–1.00)
Decision tree (trials = 10, model = rules, window = TRUE)	0.85 (0.55–0.98)	0.86	0.83	0.86	0.83	0.69	0.85 (0.63–1.00)
Neural network (size = 3, decay = 1e-04)	0.85 (0.55–0.98)	0.86	0.83	0.86	0.83	0.69	0.85 (0.63–1.00)
Support vector machines (linear kernel) (C = 1)	0.85 (0.55–0.98)	0.86	0.83	0.86	0.83	0.69	0.85 (0.63–1.00)
Support vector machines (radial kernel) (sigma = 1.432815, C = 1)	0.77 (0.46–0.95)	0.71	0.83	0.83	0.71	0.54	0.85 (0.63–1.00)
Support vector machines (polynomial kernel) (degree = 1, scale = 0.1, C = 0.5)	0.85 (0.55–0.98)	0.86	0.83	0.86	0.83	0.69	0.85 (0.63–1.00)
Random forest (mtry = 32)	0.77 (0.46–0.95)	0.71	0.83	0.83	0.71	0.54	0.90 (0.74–1.00)
Mean value (SD)	0.81 (0.04)	0.79 (0.08)	0.83 (0.00)	0.85 (0.02)	0.77 (0.06)	0.62 (0.08)	0.85 (0.04)

*PPV* positive predictive value; *NPV* negative predictive value; *AUC* area under the receiver operating curve

## Discussion

This study used an electronic nose to develop a breath test for VAP. We focused on standardizing the process of establishing machine learning algorithms. After all the procedures were standardized, the breath test developed herein had a high diagnostic accuracy in predicting VAP.

AI has gradually been applied to the medical field, especially machine learning techniques that analyze medical data to establish a prediction model. Applying machine learning techniques in medicine is a positive development. However, many defects restrict the future development of AI in medicine. First, few studies report the procedures of model selection and data processing, which makes the statistical analysis look like a “black box.” In this study, we report the procedures of model selection and data processing to enhance the transparency of the study. Second, from an epidemiological point of view, many AI researchers in medicine lack the concept of epidemiological study design and do not report the essential items for reporting diagnostic accuracy. In this study, we followed the standards for reporting of diagnostic accuracy studies (STARD) guidelines to enhance the quality of the research [38]. Third, many machine learning studies reported only the best accuracy value without showing details for readers to evaluate the reliability of test results. In this study, we carefully selected machine learning algorithms that are suitable for the learning task for classification. Because the relationships of sensor response variables were initially unclear, this study also used eight types of machine learning to provide a reliable estimation of the accuracy with the mean value. We found that the data of sensor arrays might be susceptible to multicollinearity for the high correlation between sensor responses, and the neural network had poor performance in this situation. Classification trees might be resistant to highly correlated sensor responses. Finally, AUC is an important index for the evaluation of diagnostic accuracy. However, one of the major practical drawbacks of the AUC as an index of diagnostic performance is that it summarizes the entire ROC curve, including regions that frequently are not relevant to practical applications (e.g., regions with low levels of specificity). In this study, we also applied a pAUC to prevent the statistical uncertainty of the estimation [39]. The results of the obtained accuracy reported in this study are therefore conservative but more reliable than AUC results for clinical physicians to judge the new AI technique. From the technical point of view, we suggest that researchers not show only the best results with the highest accuracy; instead, a study should clearly explain all the procedures and conservatively estimate the accuracy for physicians in making clinical decisions.

In vitro studies have reported that an electronic nose was able to detect *Staphylococcus aureus, Haemophilus influenzae, Escherichia coli, Pseudomonas aeruginosa, Moraxella catarrhalis*, *Streptococcus pneumoniae,* and *Mycobacterium tuberculosis* in bacterial cultures [40–42]. In human studies, van Geffena et al. reported that the electronic nose could discriminate bacterial and viral infections in patients with chronic obstructive pulmonary disease with acute exacerbations [43]. A study used an electronic nose to detect pulmonary aspergillosis in patients with prolonged chemotherapy-induced neutropenia and reported an accuracy of 90.9% [44]. In ventilated patients**,** Hockstein et al. used an electronic nose to diagnose VAP and showed a correlation between the electronic sensor response and the pneumonia score, with a diagnostic accuracy of 70% [45]. Schnabel et al. used an electronic nose to diagnose VAP and reported a sensitivity of 88% and a specificity of 66% [46]. Liao et al. selected 12 patients with *Pseudomonas aeruginosa* infection and 12 patients as noninfectious controls to diagnose VAP with *Pseudomonas aeruginosa* infection in the ICU with the Cyranose 320 electronic nose. The study reported accuracy of 0.95 and a positive predictive value of 0.93 but did not provide the specificity, a negative predictive value, or Kappa values [47]. In fact, in in-hospital patients, especially in the ICU, most of the bacterial cultures report many bacteria, and one single bacteria is not common. Therefore, the selection of patients may introduce selection bias that makes it difficult to generalize the research results in clinical practice. For critical patients in the ICU, physicians’ primary concern is whether frail patients have bacterial pneumonia, and then they can empirically prescribe broad-spectrum antibiotics on time. Our study did not attempt to detect one single bacteria and therefore prevented misclassification bias from selecting study subjects. From the medical point of view, we suggest that future medical AI researchers must know the need in clinical practice, and then the research can truly promote the application of AI in clinical application.

In the breath study of ICU patients, we must consider the influence of multiple comorbidities that may decrease discrimination ability. Many diseases, such as diabetes, acute renal failure, and acute hepatitis, may influence breath metabolites [48–50]. Moreover, many subjects had coinfection at other sites, such as the urinary tract and skin. Multiple infectious diseases with co-existing varied pathogens might also decrease the discrimination ability. To prevent confounding results, we suggest that further studies should consider more restrictive exclusion criteria and controls individually matched by age (+/− 5 years) and gender. Owing to a limited number of subjects, we did not conduct an independent external validation test. The results must be interpreted carefully. We suggest enrolling more study subjects in different hospitals to validate the results before clinical use.

Some AI researchers may use an independent dataset from another group of subjects to assess the accuracy in the external validation [26]. However, in fundamental knowledge of epidemiology, we know that the prevalence of the disease in the training set and testing sets will influence the accuracy of a diagnostic test. A higher prevalence of the disease in testing subjects will have a higher positive predictive value [51]. Therefore, most studies have used a ratio of 1:1 in the number of cases and controls to obtain the most optimized results, in which the prevalence of the disease is 50%. However, in clinical practice, especially for screening purposes in a community, the majority of subjects are healthy, and the prevalence of disease is low. For this reason, Leopold et al. reviewed the performance of an electronic nose during external validation. The results showed that better performance of the external validation set was always observed when subjects included in the external validation set were derived from the same population or hospitals as the training set; however, decreased performance of the external validation set was observed when the study subjects included in the external validation set were enrolled from other hospitals [52]. Therefore, researchers should know the limitation of external validation. A prediction model established from hospital patients might not be suitable for community screening. From an epidemiological point of view, we suggest that AI researchers should carefully examine their study design and select suitable study subjects in consideration of their future application.

Though the random forest algorithm had the highest AUC in this study, there are many machine learning algorithms, and most electronic nose studies tried many machine learning technique and showed the algorithm with the best accuracy. We conducted a literature search in PubMed at https://www.ncbi for “(machine learning) AND (electronic nose) NOT review [Publication Type]” published in 2019. After eliminating papers without full text or did not provide details of sensor, this search produced 17 results published in 2019. The most common used algorithm was support vector machine (10 studies), followed by neural network (6 studies), random forest (4 studies), *k*-nearest neighbor (4 studies), and then linear discrimination analysis (3 studies). Support vector machine combines both the instance-based nearest neighbor and the linear regression modeling to model highly complex relationships by creating hyperplanes [27]. SVM algorithms have been implemented in several well-supported libraries across many programming languages and exploded in popularity [27]. We have summarized the strength and weaknesses of common machine learning techniques (Table 3). For researchers who are not familiar with machine learning, we suggest that SVM algorithms might be a suitable solution to analyze the sensor array data.

**Table 3:** Comparison of strengths and weaknesses of machine learning algorithms in electronic nose studies

	Strengths	Weaknesses
*k*-nearest neighbors	• Make no assumption about underlying data distribution	• Does not produce a model, limiting the ability to understand how the features are related to the class
		• If there are more samples of one class than other class, the dominant class will control the classification and cause wrong classification
Naive Bayes	• Requires relatively few examples for training	• Relies on an often-faulty assumption of equally important and independent features
		• Not ideal for datasets with many numeric features
Decision tree	• Can be used on small dataset	• It is easy to overfit or underfit the model
	• Model is easy to interpret	• Small changes in the training data can result in large changes to decision logic
Neural network	• Conceptually similar to human neural function	• Very prone to overfitting training data
	• Capable of modeling more complex patterns	• Susceptible to multicollinearity
Support vector machines	• High accuracy but not overly influenced by noisy data and not very prone to overfitting	• Finding the best model requires testing of various combinations of kernels and model parameters
	• Easier for users due to the existence of several well-supported SVM algorithms	
	• Most commonly used	
Random forest	• Can handle noisy or missing data	• The model is not easily interpretable
	• Suitable for class imbalance problems	

Summarized from [27, 53, 54]

### Limitation

In this study, we operated the electronic nose at 20–22 degrees Celsius, which is different from the temperature of the human exhaled air of around 37 degrees Celsius. We suggest future studies use GC-MS to compare the changes in the composition of collected breath at different temperatures.

## Conclusion

An electronic nose can discriminate the distinct patterns of VOCs derived from pathogens and be applied to diagnose VAP. Using sensor arrays to analyze VOCs has potential in the development of a new screening test for VAP in the ICU. The potential of the AI technique in clinical medicine is expected but not yet fully recognized. Although it is reasonable to expect high predictive accuracy in making predictions owing to the development of increasingly elaborate machine learning algorithms, we should also advocate for further research to address the importance of epidemiological study design and strengthen the reporting of procedures to test accuracy.

## Abbreviations

AI: Artificial intelligence
ALT: Alanine aminotransferase
AST: Aspartate aminotransferase
BUN: Blood urine nitrogen
CBC: Complete blood count
GC-MS: Gas chromatography/mass spectrometry
ICU: Intensive care unit
MV: Mechanically ventilated
NPV: Negative predictive value
PPV: Positive predictive value
ROC: Receiver operator characteristic
STARD: Standards for reporting of diagnostic accuracy studies
SVMs: Support vector machines
VAP: Ventilator-associated pneumonia
VOCs: Volatile organic compounds
